# Essential Oils as Bioinsecticides Against *Blattella germanica* (Linnaeus, 1767): Evaluating Its Efficacy Under a Practical Framework

**DOI:** 10.3390/insects16010098

**Published:** 2025-01-18

**Authors:** Ana Manzanares-Sierra, Eduard Monsonís-Güell, Crisanto Gómez, Sílvia Abril, Mara Moreno-Gómez

**Affiliations:** 1 Environmental Science Department, University of Girona,17003 Girona, Spain; crisanto.gomez@udg.edu (C.G.); silvia.abril@udg.edu (S.A.); 2 Acondicionamiento Tarrasense, Carrer de la Innovació 2, 08225 Terrassa, Spain; 3 Henkel Ibérica S.A, Research and Development (R&D) Insect Control Department, 08005 Barcelona, Spainmara.moreno@henkel.com (M.M.-G.)

**Keywords:** essential oils, bioinsecticides, low-risk biocides, urban pests, *Blattella germanica*

## Abstract

Concerns about the negative effects of traditional insecticides and increasing insecticide resistance have prompted the exploration of botanical alternatives like essential oils (EOs). The registration of biocides is a mandatory procedure, and some regions have established a special status for compounds that meet specific low-risk criteria, which includes certain EOs. This study aimed to evaluate and compare the efficacy of sixteen EOs, both registered as low-risk biocides and/or standard biocides, against the German cockroach, *Blattella germanica*. A topical application was performed with undiluted EOs (15 µL) on the dorsal surface of awake cockroaches. The results showed significant variations in efficacy, as follows: thyme, sweet orange, and lavender oils were the most effective, achieving 100% mortality within 24 h and a less than 30 s knockdown effect. In contrast, linseed and cottonseed oils were the least effective, resulting in 70% and 40% 24 h mortality. Remarkably, some EOs registered as low-risk biocides performed as well as the ones registered as standard biocides, suggesting that this category does not have to always be at odds with efficacy. Future research should adopt practical, application-driven approaches to ensure bioinsecticides balance performance and safety, meeting both regulatory and consumer demands.

## Simple Summary

With rising concerns about traditional chemical insecticides, there is growing interest in safer, natural alternatives. Among these options, essential oils have received special attention. While some essential oils are registered through traditional routes for standard biocides, others are registered as low-risk biocides, as they meet specific criteria for minimal risk. This study evaluates and compares the insecticide efficacy of essential oils against the German cockroach, *Blattella germanica*, a major and widespread urban pest. Sixteen essential oils, both registered as low-risk biocides and/or standard biocides, were tested in a topical application experiment. The results showed that, while all essential oils tested were toxic to the cockroaches, their effectiveness varied. Thyme, sweet orange, and lavender oils were the most effective, achieving knockdown in less than 30 s and 100% mortality in 24 h. Linseed and cottonseed oils were the least effective, with 24 h mortality rates of 70% and 40%, respectively. Importantly, some of the oils classified as low-risk were just as effective as others classified as standard biocides, thus suggesting that low-risk classification does not necessarily compromise efficacy. These findings underscore the potential of essential oils as bioinsecticides, emphasizing the importance of considering both insecticidal performance with low-risk attributes.

## 1. Introduction

The public’s increasing concern about the potentially negative effects of traditional insecticides, along with the growing restrictions on their use, has motivated the development of botanical alternatives [1]. Furthermore, numerous studies have documented resistance to commercial insecticides, driving further research into novel solutions [2,3]. Among bioinsecticides, natural products containing essential oils (EOs) and essential oil components (EOCs) have enjoyed the most attention, owing to widespread reports of activity [4]. The intrinsic properties of EOs interfere with the basic metabolic, biochemical, and physiological functions of insect pests [5]. Several studies have demonstrated that EOs induce neurotoxic effects, leading to the paralysis and subsequent death of insects [6]. Among the various action mechanisms, the inhibition of acetylcholinesterase, interference with the octopaminergic system, and modulation of GABA-gated chloride channels are some of the most widely investigated neurotoxic effects of EOs [5,7]. Despite the extensive literature on the insecticidal activity of EOs [4,8,9,10], the majority of studies focus on agricultural and post-harvest pests [10,11,12], with fewer targeting urban pests [9,13]. Moreover, variations in methodologies, target species, developmental stages, oil composition, and the diverse ways results are presented make it difficult to draw conclusions [4].

When developing bioinsecticides, it is crucial to consider several factors apart from their level of biological efficacy [14]. The availability and price of compounds are two important factors that are closely intertwined. Commonly used substances found in a range of products (fragrances, cleaning agents, condiments, etc.) are usually manufactured on a large scale, leading to regulated market prices. However, for uncommon compounds, prices may become excessively high for pest control purposes [15]. Nevertheless, the final obstacle to the commercialization of bioinsecticides is obtaining registration approval, which is strictly regulated by competent authorities in each region [14]. The registration process of an insecticide is mandatory and widely recognized as one of the primary challenges currently hindering the introduction of new bioinsecticides to the market [4,15,16,17]. For this reason, some regions with stricter regulations have established a special status from regulatory agencies for some compounds that meet specific criteria for minimal risk [18,19,20]. Subsequently, some EOs have been categorized as low-risk biocides (LRB) in some regions, benefiting from regulatory advantages for incorporation into insecticide products [9]. Biocide regulations can vary significantly between different countries and regions [21]. A compound that is approved in one region may not be approved in another, due to differences in regulatory requirements and risk assessments [16]. Also, the registration category could be different for the same compound in different regions. As a result, some EOs are categorized as LRB in some regions and as standard biocides (SB) in others.

In the field of urban pest control, contact insecticides have become a valuable tool [22,23]. These insecticides are based on topical toxicity and can be applied as surface treatments or through direct application, depending on the objective and the product format [24]. EOs penetrate the insect cuticle effectively, increasing their own bioavailability inside insect pests and, therefore, their toxicity [25]. This makes them good candidates for incorporation into contact insecticides [26,27,28]. However, many EOs are known for their high volatility, as many of their EOCs evaporate soon after application, resulting in low persistence [29]. This property has been pointed out as a concern in their practical use, because it reduces the residual effects of application [7]. On the other hand, the low persistence also makes EOs safer, as it minimizes the risk of affecting non-target organisms and the environment [29]. For many common EOs, their safety for humans is relatively well documented and understood, and they tend to be relatively non-toxic to birds, fish, and other wildlife [16,17].

Among urban pests, the German cockroach, *Blattella germanica* (Linnaeus, 1767), is considered one of the major pests worldwide [30,31,32]. *B. germanica* infests indoor environments and is recognized as a public health pest, causing economic losses and endangering human health [31,32,33]. The current control strategies primarily rely on synthetic insecticides [34]. However, the widespread resistance to many of these insecticides is a pervasive concern, necessitating continued innovation to reduce reliance on potential hazardous treatments [34,35,36].

This study aimed to evaluate and compare the effectiveness of EOs, both registered as SB and LRB, to be incorporated into contact insecticide products against *B. germanica*.

## 2. Materials and Methods

This study was conducted at the laboratory of Henkel-R&D International Insect Control category (Spain) and focuses on registered EOs in areas with stricter biocide product regulations, Europe (EU), the United States (USA), and South Korea (KOR) (Regnault-Roger, Vincent, and Arnason 2012).

### 2.1. Insects

A long-established non-resistant laboratory strain of *B. germanica* was used. The colonies are maintained in the laboratory of Henkel-R&D International Insect Control category at 25 ± 2 °C, 60 ± 5% RH, and 12:12 h (L:D) photoperiod. Water and dog chow were provided ad libitum. The test was performed with non-gravid females of *B. germanica*, because it has been demonstrated that they are the most difficult life stage to control due to their larger body size and fat composition [1,37].

### 2.2. Essential Oils

Two registration categories of EOs have been tested: LRB and SB. Twelve LRB registered EOs were tested against *B. germanica* (Table 1). These oils were selected from among the 21 included in the following lists: Annex 1, 2012 of BPR [38], Active Ingredients Eligible for FIFRA 25(b) Pesticide Products [39] and the list of low-risk biocidal active substances of Korea [40]. All of them are registered as LRB within the target regions (EU, USA, and KOR) and were selected based on organoleptic properties and sample availability. Also, redundancies among varieties of the same EO were avoided. For example, only the China variety of cedarwood was selected, due to its availability, excluding other varieties such as Virginia and Texas. Among these oils, three are registered either as LRB or conventional biocides, depending on the region, as follows: clove oil, lavender oil, and lemongrass oil. Moreover, geraniol, although not an EO, was included, as it is widely recognized for its insecticidal properties [41]. Geraniol is an EOC considered either LRB or SB, depending on the region (Table 1). Three EOs registered as SB in any of the three regions were incorporated into the analysis, as follows: oil of bergamot, sweet orange oil, and eucalyptus oil. (Table 1).

All of the compounds tested were obtained from the “Henkel Fragrance Center” department (Henkel AG & Co. KGaA, Düsseldorf, Germany). They were applied undiluted, thus at a 100% EO concentration, at a dose of 15 µL per individual cockroach. Pure EOs were used in this study to evaluate their efficacy without potential alterations from co-formulants. The dose was chosen because it represents the amount of formula that will reach an individual *B. germanica* when sprayed from a distance of 30 cm with a one-gram output trigger.

### 2.3. Topical Toxicity Assay

**Figure 1:**
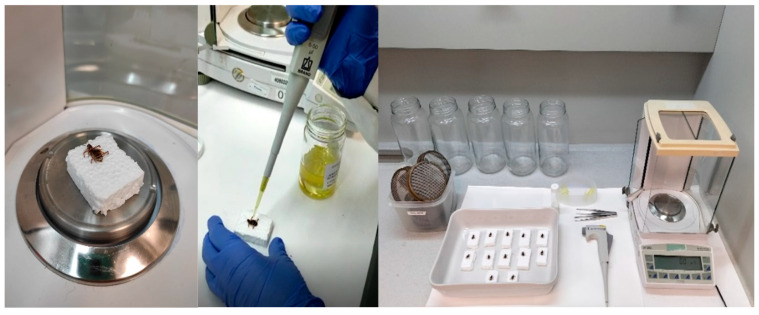
From left to right: immobilized female *Blattella germanica* on the support, application of treatment, and set of materials used for the topical toxicity assay.

A topical application test was used to determine the toxicity of the selected EOs [13]. The insects were anesthetized using carbon dioxide. The gas was supplied from a 47.5 L cylinder from Linde Gas España S.A.U. It was opened at a pressure of 50 bars for 20 s, allowing the carbon dioxide to flow into 21 L containers where the roaches were allocated. These conditions ensure that the insects are immobilized for easy handling. They were individually immobilized on expanded polystyrene supports (1 × 1.5 cm) with the dorsal area exposed. Two staples were used to immobilize them, with one under the pronotum and the other at the end of the abdominal area (Figure 1). The application was performed after 10 min, when observing that the insects had awakened from anesthesia and showed activity. Using a hand microapplicator (Transferopette^®^), 15 µL of EO was applied to the dorsal area of the insect, over the wings. After treatment, the insects were released from the support and transferred into glass jars with diameters of 10.5 cm and heights of 22.5 cm, covered with metal grid lids. The condition of the insects (alive/dead or KD) was recorded immediately after treatment, during the first 7 h (0.1, 0.5, 1, 2, 3, 4, 5, 15, 30, 60, 120, 180, 240, 300, 360, and 420 min), as well as 24 h after treatment. Ten replicates were carried out per treatment, using one individual per replicate. Additionally, 10 control replicates were performed following the same protocol without applying treatment. For the control replicates, the same handling procedure was followed, and the insects were immobilized in the same manner. No solvent, water, or any other solution was applied to the insects in the control group. All studies were conducted at 25 ± 5 °C and 50 ± 10% RH [42,43].

### 2.4. Endpoints

In assessing the insecticidal efficacy of EOs, both knockdown (KD) and mortality effects were considered. KD is the rapid paralysis of insects by an insecticide, causing them to fall down and remain in a state such as to be inapable of coordination and lie apparently dead, unable to fly or walk in a coordinated way [40]. Knockdown rate (KR%) is a measure of the percentage of insects knocked down scaled to the size of that sample. Mortality refers to dead arthropods that do not move, even when poked or probed [38]. Mortality rate (MR%) is a measure of the number of deaths in a population, scaled to the size of that sample. In this study, a correction of the mortality rates by Abbot’s formula has not been necessary, as no mortality was observed in the controls [44].

Regulatory agencies have high efficacy standards to consider a product effective. For example, the ECHA in Europe recommends up to 90% mortality after 24 h for direct-contact insecticides. In this study, a 90% mortality after 24 h threshold was fixed to consider an EO effective [42].

Determining the time required to achieve 50% knockdown in the insects (KT50) accurately was not feasible, due to the limited observation time within the initial seconds of the experiment, when most of the EOs achieved 100% KD. Therefore, the parameter used to evaluate the speed of action of EOs was the time taken to reach 50% mortality (MT50 in minutes).

### 2.5. Statistical Analysis

All statistical analyses were conducted using R version 4.1.3. [45]. The significance level alpha was 0.05 for all statistical analyses. To compare the efficacy between EOs in terms of knockdown rates and mortality rates within the observation time, generalized linear models (GLM) were performed (Quasibinomial and Binomial error distributions and logit function). A post hoc analysis to compare between pairs of EOs was performed using the pairs function of the emmeans package. The MT50 of the different EOs was calculated using GLM and the dose.p function from the MASS package. Either mortality or KD were considered as the response variable, with the treatment (different EOs applied) serving as the fixed factor, while time was included as a covariate in the analysis.

## 3. Results

**Figure 2:**
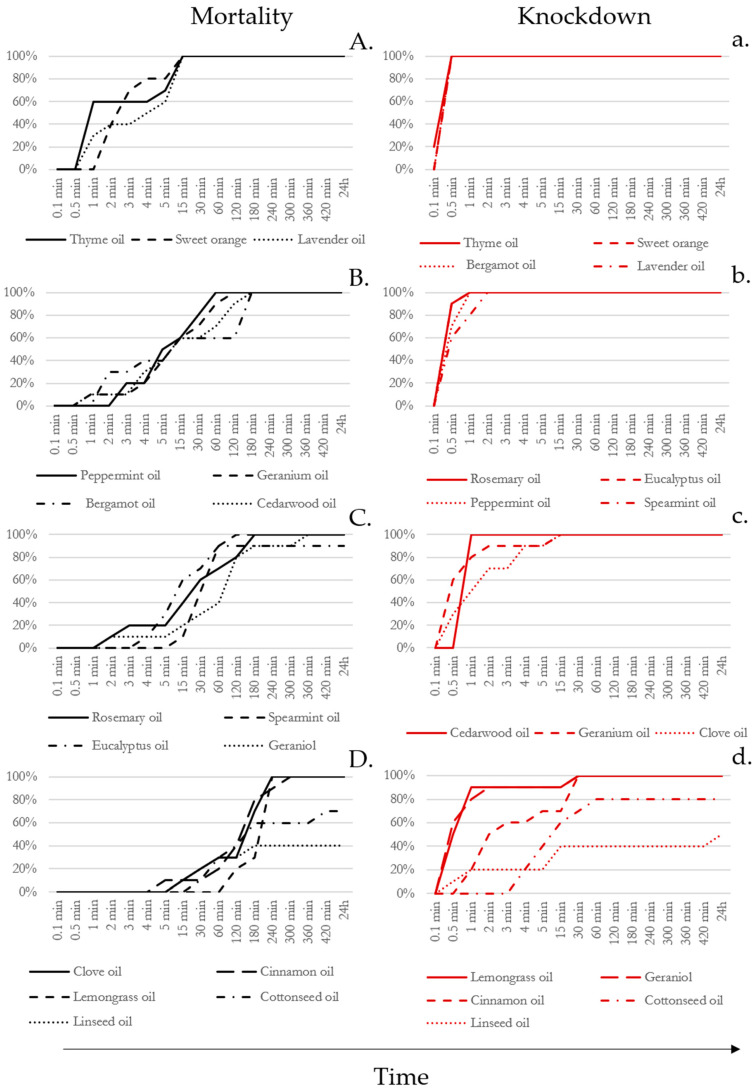
Mortality rate (MR% on the left, in uppercase) and Knockdown rate (KR% on the right, in lowercase) curves of the different EO treatments. Treatments are organized by declining efficacy: uppercase letters (**A**–**D**) represent mortality (MR%), and lowercase letters (**a**–**d**) represent knockdown (KR%).

**Figure 3:**
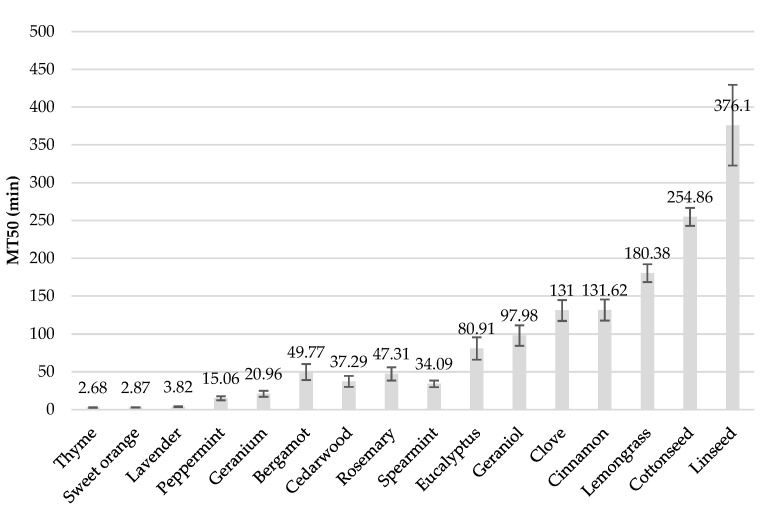
Time to reach 50% mortality in minutes (MT50 ± SE) of each EO. MT50 values were derived from the GLM analysis using the dose.p function.

No mortality was observed in any of the controls. All EOs tested exhibited toxicity towards roaches, albeit with variations in toxicological effects and speed of action (Figure 2). Within 24 h, all treatments resulted in 100% mortality, except for eucalyptus oil, cottonseed oil, and linseed oil, which achieved mortality rates of 90%, 70%, and 40%, respectively (Figure 2A–D). Eucalyptus oil reached 100% KR% at 24 h, while cottonseed oil and linseed oil only attained a KR% of 50% and 80%, respectively (Figure 2d). MT50 ranged from 2.68 ± 0.48 to 376.10 ± 53.49 min, with thyme oil demonstrating the highest effectiveness and linseed oil the lowest (Figure 3). However, calculating KT50 parameters with accuracy was not possible because of the lack of observations within the first seconds of the experiment. As illustrated in Figure 2a–d, most EOs achieved 100% KR% within the first few minutes of the experiment. Specifically, thyme oil, sweet orange oil, lavender oil, and bergamot oil achieved complete KR% within the initial 30 s.

Thyme oil, sweet orange oil, and lavender oil were the most effective EOs in terms of mortality, while linseed oil was the least effective, followed by cottonseed oil (Table 2). Regarding KD, thyme oil, orange oil, bergamot oil, lavender oil, rosemary oil, eucalyptus oil, peppermint oil, and spearmint oil exhibited the best results, being statistically equally effective (Table 2). Aligning with the observed mortality rates, the oils with the poorest performance in terms of KD were linseed oil and cottonseed oil. Statistical analysis details of the comparisons one by one, both in terms of mortality and KD, are shown in Appendix A (Table A1 and Table A2). In this study, it has been observed that, overall, there is a correspondence between KD time and mortality; moreover, oils with shorter KD times generally had quicker mortality (Figure 2 and Table 2).

## 4. Discussion

The observed significant differences in the performance of EOs regarding both mortality and KD effects highlight the variable efficacy of these compounds. Thyme, sweet orange, and lavender oils are the most effective in both analyses, while linseed and cottonseed oils exhibited comparatively lower effectiveness across both parameters.

Interestingly, EOs classified as SB did not inherently demonstrate greater effectiveness compared to those EOs categorized as LRB. This observation underscores an important consideration in the development of bioinsecticides, suggesting that EO efficacy is not necessarily compromised by the low-risk categorization. Geraniol, an EOC recognized as SB in Europe and South Korea and as LRB in the USA, is commonly included in various commercial botanical pesticide products [14,15]. However, it was not among the most effective compounds tested in this study. Geranium oil, one of whose main constituents is geraniol, demonstrated better efficacy in terms of mortality [8,46]. This result aligns with observations made by other authors who have noted that the complexity of EOs often leads to better outcomes than those achieved by their individual components [14,17,47,48,49]. EOs are often complex mixtures of compounds (terpenoids, aldehydes, esters, etc.), where the overall bioactivity frequently results from synergy among these constituents [14,17,47,49]. Goharrostami et al. (2022) found that thyme oil was the most toxic substance in a study evaluating the topical toxicity of thyme oil versus its two main components (thymol and carvacrol) [49]. The synergy among EO terpenoids was also demonstrated in a study with rosemary oil, where the mechanism of action was suggested to be enhanced penetration due to the mix of EOCs [47]. In this study, single EOs have been tested, but mixtures of EOs were not evaluated. Mixed EO combinations did not necessarily yield better results in previous studies [48,50,51]. Gillian (2012) observed that mixed EO combinations did not alter their activity in topical assays against the American cockroach, *Periplaneta americana* (Linnaeus, 1758). Given the significant price differences among EOs, it is important to identify the most effective individual oils and determine whether the performance of a formulation can be improved by adding additional EOs [48,51]. In this study, EOs were tested in their pure form but, in commercial bioinsecticides, they are combined with co-formulants [14]. These co-formulants, such as silicone and paraffin oils, can improve both the toxicity and the persistence or rapid action of the EOs [52,53,54,55]. For example, the bioinsecticide EcoRaider demonstrated an additional slow-killing effect and high efficacy, likely due to the interaction between EOs and co-formulants. One proposed mechanism was that surfactants in the formulation damage the wax layer of the insect cuticle, facilitating the penetration of EOs through the cuticle [56]. Further studies should focus on the interaction between EOs and co-formulants in insecticide formulations to maximize their efficacy.

The methodological variability in studies assessing the insecticidal activity of EOs presents challenges in making consistent comparisons across the literature [4]. Each study often uses different materials, approaches, and parameters, which complicates drawing consistent conclusions from the literature. Therefore, the only reliable comparisons can often be made within the same study or when testing other oils under the same conditions to evaluate their efficacy. The comparative analysis conducted has enabled the classification of sixteen commercially available EOs, with the potential for biocide registration, based on their topical toxicity against *B. germanica*. Previous investigations assessing topical toxicity have typically applied EO solutions in acetone between the metathoracic legs of cockroaches [1,13,57], not always accounting for the potential solvent’s effects or the specific application area. This contrasts significantly with actual field conditions, where insecticides are typically sprayed onto the dorsal surface of awake cockroaches. The cuticle is the first and major barrier, protecting the insect from penetration of external compounds, and their properties vary across insects’ bodies [58,59]. As a result, the efficacy of a treatment may be affected by the application site. Here, both the amount of product (common in commercial sprayers) and the condition of the insect upon spraying (i.e., awake insect with exposed dorsum) have been considered. Although the dose of EO applied was higher than that found in commercial products, which never contain 100% EOs, this approach allowed us to detect differences in efficacy among the tested EOs [12,14]. It also revealed that certain oils, such as linseed and cottonseed oil, would not achieve 100% mortality even a concentration of 100%.

Another aspect explored in our study is the inclusion of two evaluation parameters: KD and mortality. Many studies on insecticidal activity against crawling insects focus solely on the mortality effect [54,60,61]. However, some products, such as cockroach sprays, are typically designed for rapid action [42]. Users expect to see insects fall immediately after spraying. Therefore, KD time is a critical parameter for evaluating the performance of a direct application product, as it indicates how quickly the product works. Although the KD state usually implies subsequent death, this does not always occur, and the insect may recover [61]. Our results indicate a relationship between both effects, with EOs that demonstrate greater efficacy in terms of KD tending to be more effective in terms of mortality.

In a study by Isman (2000) to determine the insecticidal activity of EOs, it was observed that the degree of toxicity of these compounds largely depended on the test species. The most interesting aspect is that there was little overlap between insect species regarding the most toxic oils and components, indicating that, although these substances are generally active against a wide range of pests, the interspecific toxicity of oils and individual compounds is highly idiosyncratic [10]. Sarac and Tunc (1995), investigating the killing action of four EOs against three species of stored-product pests, reached the same conclusion [62]. In this study, *B. germanica* was the target species, as it is one of the major indoor sanitary pests worldwide [63]. However, further studies should be conducted to explore the specific susceptibility of other urban pests to the targeted EOs.

With the rising public concern regarding the safety and efficacy of traditional insecticides, the investigation of EOs classified as low-risk biocides reveals an alternative that does not inherently compromise performance. This approach, which balances insecticidal efficacy with low-risk attributes, could contribute to the development of safer, more sustainable pest control solutions. Future studies should aim to refine the understanding of how EOs, particularly low-risk options, can be optimized for practical applications in pest management.

## Figures and Tables

**Table 1:** Compounds used in the topical toxicity assay, including their registration status and their Chemical Abstracts Service (CAS) number.

	Compound	Registration Status ^1^			CAS
		UE	USA	KOR	
Low-Risk Biocides	Cedarwood oil (China)	NR	LRB	NR	85085-29-6
	Cinnamon oil	NR	LRB	NR	8015-91-6
	Clove oil	NR	LRB	SB	8000-34-8
	Cottonseed oil	NR	LRB	NR	8001-29-4
	Geraniol	SB	LRB	SB	106-24-1
	Geranium oil	NR	LRB	NR	8000-46-2
	Lavender oil	LRB	SB	LRB	8000-28-0
	Lemongrass oil	NR	LRB	SB	8007-02-1
	Linseed oil	LRB	LRB	LRB	8001-26-1
	Peppermint oil	LRB	LRB	LRB	8006-90-4
	Rosemary oil	NR	LRB	NR	8000-25-7
	Spearmint oil	NR	LRB	NR	8008-79-5
	Thyme oil	NR	LRB	NR	8007-46-3
Standard Biocides	Bergamot oil	NR	SB	NR	8007-75-8
	Eucalyptus oil	NR	SB	SB	8000-48-4
	Sweet orange oil	SB	NR	SB	8028-48-6

^1^ Biocide registration status in different study areas: low-risk biocide (LRB), standard biocide (SB), or not registered (NR).

**Table 2:** Statistical comparison of MR% and KR% curves of the different EO treatments. EOs with the same letter are not significantly different at the α = 0.05 level according to the GLM post hoc test performed using pairs function of the emmeans package.

	Thyme	Sweet orange	Lavender	Peppermint	Geranium	Bergamot	Cedarwood	Rosemary	Spearmint	Eucalyptus	Geraniol	Clove	Cinnamon	Lemongrass	Cottonseed	Linseed
Mortality	a	a	a	b	b	b	b	bc	bc	bc	cd	de	de	ef	fg	g
KD	a	ab	ab	abc	bcd	ab	bcd	abc	abc	abc	cd	d	e	cd	f	g

## Data Availability

The datasets generated during and/or analyzed during this study are available from the corresponding author upon reasonable request.

## Appendix A

**Table A1:** Statistical analysis (GLM with post hoc test using the pairs function from the emmeans package) showing pairwise comparisons of EOs in terms of KR%. Significance level set at α = 0.05.

Comparison	Estimate	SE	z.ratio	*p*-Value
Bergamot-Cedarwood	0.80	0.41	1.94	0.05
Bergamot-Cinnamon	2.02	0.38	5.25	0.00
Bergamot-Clove	1.28	0.40	3.23	0.00
Bergamot-Cottonseed	3.06	0.39	7.84	0.00
Bergamot-Eucalyptus	0.11	0.46	0.23	0.82
Bergamot-Geraniol	0.86	0.41	2.09	0.04
Bergamot-Geranium	0.80	0.41	1.94	0.05
Bergamot-Lavender	0.00	0.47	0.00	1.00
Bergamot-Lemongrass	0.86	0.41	2.09	0.04
Bergamot-Linseed	4.38	0.42	10.51	0.00
Bergamot-Peppermint	0.29	0.45	0.66	0.51
Bergamot-Rosemary	0.11	0.46	0.23	0.82
Bergamot-Spearmint	0.53	0.43	1.25	0.21
Bergamot-Sweet orange	0.00	0.47	0.00	1.00
Bergamot-Thyme	−0.24	0.50	−0.49	0.62
Cedarwood-Cinnamon	1.21	0.31	3.87	0.00
Cedarwood-Clove	0.48	0.33	1.46	0.15
Cedarwood-Cottonseed	2.25	0.32	7.03	0.00
Cedarwood-Eucalyptus	−0.70	0.40	−1.73	0.08
Cedarwood-Geraniol	0.06	0.35	0.17	0.86
Cedarwood-Geranium	0.00	0.35	0.00	1.00
Cedarwood-Lavender	−0.80	0.41	−1.94	0.05
Cedarwood-Lemongrass	0.06	0.35	0.17	0.86
Cedarwood-Linseed	3.58	0.35	10.15	0.00
Cedarwood-Peppermint	−0.51	0.39	−1.32	0.19
Cedarwood-Rosemary	−0.70	0.40	−1.73	0.08
Cedarwood-Spearmint	−0.27	0.37	−0.73	0.47
Cedarwood-Sweet orange	−0.80	0.41	−1.94	0.05
Cedarwood-Thyme	−1.05	0.44	−2.36	0.02
Cinnamon-Clove	−0.73	0.29	−2.53	0.01
Cinnamon-Cottonseed	1.04	0.28	3.74	0.00
Cinnamon-Eucalyptus	−1.91	0.37	−5.13	0.00
Cinnamon-Geraniol	−1.15	0.31	−3.72	0.00
Cinnamon-Geranium	−1.21	0.31	−3.87	0.00
Cinnamon-Lavender	−2.02	0.38	−5.25	0.00
Cinnamon-Lemongrass	−1.15	0.31	−3.72	0.00
Cinnamon-Linseed	2.37	0.31	7.57	0.00
Cinnamon-Peppermint	−1.72	0.35	−4.88	0.00
Cinnamon-Rosemary	−1.91	0.37	−5.13	0.00
Cinnamon-Spearmint	−1.48	0.33	−4.46	0.00
Cinnamon-Sweet orange	−2.02	0.38	−5.25	0.00
Cinnamon-Thyme	−2.26	0.41	−5.45	0.00
Clove-Cottonseed	1.77	0.30	5.98	0.00
Clove-Eucalyptus	−1.18	0.39	−3.05	0.00
Clove-Geraniol	−0.42	0.33	−1.29	0.20
Clove-Geranium	−0.48	0.33	−1.46	0.15
Clove-Lavender	−1.28	0.40	−3.23	0.00
Clove-Lemongrass	−0.42	0.33	−1.29	0.20
Clove-Linseed	3.10	0.33	9.37	0.00
Clove-Peppermint	−0.99	0.37	−2.69	0.01
Clove-Rosemary	−1.18	0.39	−3.05	0.00
Clove-Spearmint	−0.75	0.35	−2.15	0.03
Clove-Sweet orange	−1.28	0.40	−3.23	0.00
Clove-Thyme	−1.53	0.43	−3.57	0.00
Cottonseed-Eucalyptus	−2.95	0.38	−7.81	0.00
Cottonseed-Geraniol	−2.19	0.32	−6.92	0.00
Cottonseed-Geranium	−2.25	0.32	−7.03	0.00
Cottonseed-Lavender	−3.06	0.39	−7.84	0.00
Cottonseed-Lemongrass	−2.19	0.32	−6.92	0.00
Cottonseed-Linseed	1.32	0.31	4.27	0.00
Cottonseed-Peppermint	−2.76	0.36	−7.69	0.00
Cottonseed-Rosemary	−2.95	0.38	−7.81	0.00
Cottonseed-Spearmint	−2.52	0.34	−7.44	0.00
Cottonseed-Sweet orange	−3.06	0.39	−7.84	0.00
Cottonseed-Thyme	−3.30	0.42	−7.85	0.00
Eucalyptus-Geraniol	0.76	0.40	1.89	0.06
Eucalyptus-Geranium	0.70	0.40	1.73	0.08
Eucalyptus-Lavender	−0.11	0.46	−0.23	0.82
Eucalyptus-Lemongrass	0.76	0.40	1.89	0.06
Eucalyptus-Linseed	4.28	0.41	10.53	0.00
Eucalyptus-Peppermint	0.19	0.43	0.43	0.67
Eucalyptus-Rosemary	0.00	0.45	0.00	1.00
Eucalyptus-Spearmint	0.43	0.42	1.02	0.31
Eucalyptus-Sweet orange	−0.11	0.46	−0.23	0.82
Eucalyptus-Thyme	−0.35	0.49	−0.72	0.47
Geraniol-Geranium	−0.06	0.35	−0.17	0.86
Geraniol-Lavender	−0.86	0.41	−2.09	0.04
Geraniol-Lemongrass	0.00	0.34	0.00	1.00
Geraniol-Linseed	3.52	0.35	10.07	0.00
Geraniol-Peppermint	−0.57	0.38	−1.48	0.14
Geraniol-Rosemary	−0.76	0.40	−1.89	0.06
Geraniol-Spearmint	−0.33	0.36	−0.90	0.37
Geraniol-Sweet orange	−0.86	0.41	−2.09	0.04
Geraniol-Thyme	−1.11	0.44	−2.51	0.01
Geranium-Lavender	−0.80	0.41	−1.94	0.05
Geranium-Lemongrass	0.06	0.35	0.17	0.86
Geranium-Linseed	3.58	0.35	10.15	0.00
Geranium-Peppermint	−0.51	0.39	−1.32	0.19
Geranium-Rosemary	−0.70	0.40	−1.73	0.08
Geranium-Spearmint	−0.27	0.37	−0.73	0.47
Geranium-Sweet orange	−0.80	0.41	−1.94	0.05
Geranium-Thyme	−1.05	0.44	−2.36	0.02
Lavender-Lemongrass	0.86	0.41	2.09	0.04
Lavender-Linseed	4.38	0.42	10.51	0.00
Lavender-Peppermint	0.29	0.45	0.66	0.51
Lavender-Rosemary	0.11	0.46	0.23	0.82
Lavender-Spearmint	0.53	0.43	1.25	0.21
Lavender-Sweet orange	0.00	0.47	0.00	1.00
Lavender-Thyme	−0.24	0.50	−0.49	0.62
Lemongrass-Linseed	3.52	0.35	10.07	0.00
Lemongrass-Peppermint	−0.57	0.38	−1.48	0.14
Lemongrass-Rosemary	−0.76	0.40	−1.89	0.06
Lemongrass-Spearmint	−0.33	0.36	−0.90	0.37
Lemongrass-Sweet orange	−0.86	0.41	−2.09	0.04
Lemongrass-Thyme	−1.11	0.44	−2.51	0.01
Linseed-Peppermint	−4.09	0.39	−10.52	0.00
Linseed-Rosemary	−4.28	0.41	−10.53	0.00
Linseed-Spearmint	−3.85	0.37	−10.41	0.00
Linseed-Sweet orange	−4.38	0.42	−10.51	0.00
Linseed-Thyme	−4.62	0.45	−10.37	0.00
Peppermint-Rosemary	−0.19	0.43	−0.43	0.67
Peppermint-Spearmint	0.24	0.40	0.60	0.55
Peppermint-Sweet orange	−0.29	0.45	−0.66	0.51
Peppermint-Thyme	−0.54	0.47	−1.14	0.25
Rosemary-Spearmint	0.43	0.42	1.02	0.31
Rosemary-Sweet orange	−0.11	0.46	−0.23	0.82
Rosemary-Thyme	−0.35	0.49	−0.72	0.47
Spearmint-Sweet orange	−0.53	0.43	−1.25	0.21
Spearmint-Thyme	−0.78	0.46	−1.70	0.09
Sweet orange-Thyme	−0.24	0.50	−0.49	0.62

**Table A2:** Statistical analysis (GLM with post hoc test using the pairs function from the emmeans package) showing pairwise comparisons of EOs in terms of MR%. Significance level set at α = 0.05.

Comparison	Estimate	SE	z.ratio	*p*-Value
Bergamot-Cedarwood	0.09	0.45	0.20	0.84
Bergamot-Cinnamon	1.91	0.56	3.43	0.00
Bergamot-Clove	1.91	0.56	3.43	0.00
Bergamot-Cottonseed	3.66	0.67	5.42	0.00
Bergamot-Eucalyptus	0.37	0.47	0.80	0.43
Bergamot-Geraniol	1.03	0.50	2.07	0.04
Bergamot-Geranium	−0.13	0.45	−0.29	0.77
Bergamot-Lavender	−0.98	0.44	−2.21	0.03
Bergamot-Lemongrass	2.98	0.63	4.73	0.00
Bergamot-Linseed	4.91	0.74	6.67	0.00
Bergamot-Peppermint	−0.21	0.45	−0.48	0.63
Bergamot-Rosemary	0.27	0.46	0.59	0.55
Bergamot-Spearmint	0.63	0.48	1.32	0.19
Bergamot-Sweet orange	−1.18	0.44	−2.65	0.01
Bergamot-Thyme	−1.34	0.45	−3.00	0.00
Cedarwood-Cinnamon	1.83	0.56	3.26	0.00
Cedarwood-Clove	1.83	0.56	3.26	0.00
Cedarwood-Cottonseed	3.57	0.68	5.28	0.00
Cedarwood-Eucalyptus	0.28	0.47	0.60	0.55
Cedarwood-Geraniol	0.95	0.50	1.88	0.06
Cedarwood-Geranium	−0.22	0.45	−0.49	0.63
Cedarwood-Lavender	−1.07	0.44	−2.40	0.02
Cedarwood-Lemongrass	2.90	0.63	4.58	0.00
Cedarwood-Linseed	4.82	0.74	6.55	0.00
Cedarwood-Peppermint	−0.30	0.45	−0.68	0.50
Cedarwood-Rosemary	0.19	0.46	0.40	0.69
Cedarwood-Spearmint	0.54	0.48	1.12	0.26
Cedarwood-Sweet orange	−1.27	0.45	−2.83	0.00
Cedarwood-Thyme	−1.43	0.45	−3.18	0.00
Cinnamon-Clove	0.00	0.63	0.00	1.00
Cinnamon-Cottonseed	1.74	0.71	2.46	0.01
Cinnamon-Eucalyptus	−1.54	0.57	−2.72	0.01
Cinnamon-Geraniol	−0.88	0.59	−1.49	0.14
Cinnamon-Geranium	−2.04	0.56	−3.67	0.00
Cinnamon-Lavender	−2.89	0.55	−5.22	0.00
Cinnamon-Lemongrass	1.07	0.68	1.58	0.11
Cinnamon-Linseed	2.99	0.75	3.97	0.00
Cinnamon-Peppermint	−2.13	0.56	−3.83	0.00
Cinnamon-Rosemary	−1.64	0.56	−2.90	0.00
Cinnamon-Spearmint	−1.29	0.57	−2.24	0.03
Cinnamon-Sweet orange	−3.09	0.56	−5.56	0.00
Cinnamon-Thyme	−3.26	0.56	−5.82	0.00
Clove-Cottonseed	1.74	0.71	2.46	0.01
Clove-Eucalyptus	−1.54	0.57	−2.72	0.01
Clove-Geraniol	−0.88	0.59	−1.49	0.14
Clove-Geranium	−2.04	0.56	−3.67	0.00
Clove-Lavender	−2.89	0.55	−5.22	0.00
Clove-Lemongrass	1.07	0.68	1.58	0.11
Clove-Linseed	2.99	0.75	3.97	0.00
Clove-Peppermint	−2.13	0.56	−3.83	0.00
Clove-Rosemary	−1.64	0.56	−2.90	0.00
Clove-Spearmint	−1.29	0.57	−2.24	0.03
Clove-Sweet orange	−3.09	0.56	−5.56	0.00
Clove-Thyme	−3.26	0.56	−5.82	0.00
Cottonseed-Eucalyptus	−3.29	0.68	−4.84	0.00
Cottonseed-Geraniol	−2.62	0.69	−3.80	0.00
Cottonseed-Geranium	−3.79	0.67	−5.62	0.00
Cottonseed-Lavender	−4.63	0.67	−6.87	0.00
Cottonseed-Lemongrass	−0.67	0.73	−0.92	0.36
Cottonseed-Linseed	1.25	0.77	1.63	0.10
Cottonseed-Peppermint	−3.87	0.67	−5.75	0.00
Cottonseed-Rosemary	−3.38	0.68	−4.99	0.00
Cottonseed-Spearmint	−3.03	0.68	−4.44	0.00
Cottonseed-Sweet orange	−4.84	0.68	−7.14	0.00
Cottonseed-Thyme	−5.00	0.68	−7.35	0.00
Eucalyptus-Geraniol	0.66	0.51	1.30	0.19
Eucalyptus-Geranium	−0.50	0.46	−1.08	0.28
Eucalyptus-Lavender	−1.35	0.46	−2.96	0.00
Eucalyptus-Lemongrass	2.61	0.64	4.10	0.00
Eucalyptus-Linseed	4.54	0.74	6.15	0.00
Eucalyptus-Peppermint	−0.59	0.46	−1.27	0.20
Eucalyptus-Rosemary	−0.10	0.47	−0.20	0.84
Eucalyptus-Spearmint	0.26	0.49	0.53	0.60
Eucalyptus-Sweet orange	−1.55	0.46	−3.38	0.00
Eucalyptus-Thyme	−1.71	0.46	−3.71	0.00
Geraniol-Geranium	−1.16	0.50	−2.34	0.02
Geraniol-Lavender	−2.01	0.49	−4.09	0.00
Geraniol-Lemongrass	1.95	0.65	2.99	0.00
Geraniol-Linseed	3.87	0.74	5.20	0.00
Geraniol-Peppermint	−1.25	0.50	−2.52	0.01
Geraniol-Rosemary	−0.76	0.51	−1.50	0.13
Geraniol-Spearmint	−0.41	0.52	−0.78	0.44
Geraniol-Sweet orange	−2.21	0.49	−4.47	0.00
Geraniol-Thyme	−2.38	0.50	−4.78	0.00
Geranium-Lavender	−0.85	0.44	−1.93	0.05
Geranium-Lemongrass	3.11	0.63	4.95	0.00
Geranium-Linseed	5.04	0.74	6.85	0.00
Geranium-Peppermint	−0.08	0.44	−0.19	0.85
Geranium-Rosemary	0.40	0.46	0.88	0.38
Geranium-Spearmint	0.76	0.47	1.60	0.11
Geranium-Sweet orange	−1.05	0.44	−2.38	0.02
Geranium-Thyme	−1.21	0.44	−2.73	0.01
Lavender-Lemongrass	3.96	0.63	6.29	0.00
Lavender-Linseed	5.88	0.74	7.97	0.00
Lavender-Peppermint	0.76	0.44	1.75	0.08
Lavender-Rosemary	1.25	0.45	2.77	0.01
Lavender-Spearmint	1.61	0.47	3.43	0.00
Lavender-Sweet orange	−0.20	0.43	−0.47	0.64
Lavender-Thyme	−0.37	0.44	−0.84	0.40
Lemongrass-Linseed	1.92	0.76	2.53	0.01
Lemongrass-Peppermint	−3.20	0.63	−5.09	0.00
Lemongrass-Rosemary	−2.71	0.64	−4.27	0.00
Lemongrass-Spearmint	−2.36	0.64	−3.67	0.00
Lemongrass-Sweet orange	−4.16	0.63	−6.59	0.00
Lemongrass-Thyme	−4.33	0.64	−6.81	0.00
Linseed-Peppermint	−5.12	0.74	−6.97	0.00
Linseed-Rosemary	−4.63	0.74	−6.28	0.00
Linseed-Spearmint	−4.28	0.74	−5.78	0.00
Linseed-Sweet orange	−6.09	0.74	−8.21	0.00
Linseed-Thyme	−6.25	0.74	−8.40	0.00
Peppermint-Rosemary	0.49	0.46	1.07	0.28
Peppermint-Spearmint	0.84	0.47	1.78	0.07
Peppermint-Sweet orange	−0.96	0.44	−2.20	0.03
Peppermint-Thyme	−1.13	0.44	−2.55	0.01
Rosemary-Spearmint	0.35	0.49	0.73	0.47
Rosemary-Sweet orange	−1.45	0.45	−3.20	0.00
Rosemary-Thyme	−1.62	0.46	−3.53	0.00
Spearmint-Sweet orange	−1.81	0.47	−3.84	0.00
Spearmint-Thyme	−1.97	0.47	−4.16	0.00
Sweet orange-Thyme	−0.17	0.44	−0.38	0.71
